# Treatment of Scarlatina

**Published:** 1862-12

**Authors:** T. Hillier


### TREATMENT OF SCARLATINA.

By T. HILLIER, M. D.

In estimating the effects of treatment, it is very important to remember that in this, as in many other diseases, some cases are so severe that no treatment will prevent death; that other cases are so mild that they will recover even if left to themselves, or badly treated; whilst a third set of cases of intermediate severity, if well treated, will recover, and the speed and completeness of their recovery will be influenced by the mode of treatment; but the same cases, if injudiciously treated, will not recover at all, or recovery will occur only after more protracted illness, and with greater damage to the constitution.

Fully to judge of the value of our treatment, we ought to know how similar cases progress if left to themselves; this our homœopathic neighbors, if they practice as they profess, might give us an opportunity of observing. We may, of course, learn much by comparing cases treated on one plan with others treated on a different plan; but here, too, we must be careful to compare cases of equal severity, and as nearly as possible alike. We have not arrived at any specific antidote to the poison of scarlatina by which we can either prevent its taking effect, or counteract its influence when in operation. As a preventive belladonna has been vaunted, but it has not stood the test of careful experiment. We know of no means of prevention but the avoidance of exposure to the infection.

This disease is frequently spread in public schools by children convalescent from the disease returning too soon after their illness to associate with other children; it is also not uncommonly spread by patients conveyed in cabs and other public vehicles. Medical men should always wash their hands after touching a case of scarlatina, and, if possible, walk some time in the open air, and change their coats, before seeing other children.

Some future Jenner may discover that the inoculation of a disease bearing the same analogy to scarlatina which cowpox does to smallpox, will prove a protection against it. At present all we can do is to combat symptoms as they arise, and help Nature to get rid of the poison. Mild cases of scarlatina require but little medical treatment. The patient should be kept in bed for a fortnight, and not allowed to leave his room for three weeks; the diet should be bland and simple (animal food being forbidden); the bowels may be regulated by mild aperients; the skin may be sponged frequently with tepid water; and the bedroom should be kept cool and well ventilated. Some simple saline medicine may be given. The plan adopted in this hospital instead of sponging, has been to anoint the whole surface twice a-day with suet. It is grateful to the patient, relieving the sense of tingling and burning heat which is frequently complained of. During convalescence the use of warm baths is indicated. If desquamation is active the hot-air bath is very useful in combination with warm water baths. This measure is especially desirable if there be albumen in the urine and anasarca.

In severer cases, quite at the outset an emetic of ipecacuanha should be given, and if the tongue be much coated an aperient dose of rhubarb and sulphate of potash may follow at the end of twelve hours. In the malignant cases, with much depression and sensorial disturbance from the first, the emetic and aperient should be omitted. In these cases, I believe, the best medicine to give is the sesquicarbonate of ammonia; and to promote the action of the skin, it may be combined with a good dose, one or two drachms, of liq. ammonia acetatis. The carbonate of ammonia may be given in doses of two, three, four, or five grains, according to the age of the child, and with frequency proportioned to the severity of the case.

In coses where the nervous symptoms predominate, such as delirium and convulsions, and the heat of the skin is very great, a remedy has been recommended, which I have only tried once, but on that occasion it certainly seemed to do good. It was first recommended by Currie, and has since been occasionally praised by others; it is spoken of very favorably by M. Trousseau. I refer to cold affusions. They have been less frequently resorted to than they probably otherwise would have been, owing to the apparent boldness of the practice, and its running counter to popular prejudice on the subject. Trousseau says: "For a long time I have employed these affusions. I declare to you that I have never administered them without deriving from them some benefit. I do not pretend that all my patients have been cured; those who have died have experienced temporary relief; the affusion, far from being injurious to them, has always moderated the symptoms, and has always appeared to retard the fatal issue." He then quotes several very severe cases in which this practice was attended with apparently the greatest benefit, and the patients made favorable recoveries.

His mode of proceeding is as follows: The patient is placed in an empty bath, and there are thrown on him three or four pails of water of a temperature from 68° to 77° Fahr. The patient is immediately afterwards wrapped in bedclothes, and placed on the bed without being wiped, but suitably covered up. Generally reaction is established before fifteen or twenty minutes have elapsed. The affusions are renewed once or twice in twenty-four hours, according to the gravity of the symptoms.

The benefits derived from this treatment, according to Trousseau, are a reduction of the frequency of the pulse, a lowering of temperature, a cessation of delirium, and a diminution of the patient's restlessness and general distress. He also states that it usually renders the eruption more vivid, and never has the effect of "striking in" the rash, as it is called.

In the case in which I employed it there had been delirium and restlessness, and great heat of skin; there was no rash. The affusion was practised with water at a temperature of about 80°. The patient expressed herself as feeling much more comfortable a few minutes after the treatment; the temperature three hours later was nearly as high as before, but the skin did not feel so pungent or dry. From this time the patient continued to improve without an unfavorable symptom. It is true that the treatment was here applied about a date when improvement frequently occurs—namely, on the sixth day of the disease; but I confess that I have not often seen a case suddenly take such a decidedly favorable turn on any other plan of treatment. I intend, when another suitable case presents itself, to make a further trial of this remedy. As originally practised by Currie, the water used was of a lower temperature (the coldest he could procure), and affusion was repeated every two or three hours if the patient's symptoms called for it. Where the heat of skin was not so great, tepid water was used. This remedy is not applicable to cases where reaction is not well established, as in cases of extreme malignancy. The wet sheet I have tried in two cases, with satisfactory results.

As the fever subsides, and the patient is left languid with a pulse soft and slower, and skin cool, quinine is very useful.

The throat, if much involved, will require treatment. For mild cases a gargle of lemon juice and honey is agreeable and useful. For redness and swelling the use of chlorate of potash in solution (eight or ten grains to the ounce) is advisable, either as a gargle, or, if the child be too young, as a wash to be injected into the mouth with the patient sitting up. If there be much swelling, the employment of tannin, either by insufflation as a powder, or in solution as a wash, is beneficial. Ice may also be given with benefit. If the tint be somewhat livid and dusky, tincture of capsicum may be advantageously added to the gargle. This remedy is strongly recommended internally by some writers on malignant scarlatina. Externally it is well to keep warm-water dressing, a bran poultice, or spongio piline around the throat. If the nose and mouth become clogged, it is important that they be syringed out frequently with warm water, or soap and water, followed by a weak solution of sulphate of copper in a few days, to prevent obstruction to respiration. If the secretion is thin and acrid, lime-water is best. In some cases, where the pulse is soft and frequent, and there is much depression, it is well to give wine from the first; and very often about the fifth day, when the tongue becomes dry, and the pulse becomes weak and falls in frequency, wine will be required if it has been withheld before. The effect of stimulants on the urinary secretion should be carefully noted, and if there be albumen in the urine, and a notable reduction in the quantity of urinary solids, they must be withheld. When there is much swelling of the neck coming on after the first week of the fever, stimulants are, I believe, indicated, and poultices should be applied externally. No good has been derived in these cases from the use of leeches. If matter forms early, lancing is desirable. For albuminuria or hæmaturia, with or without dropsy at the commencement, the great indication is to obtain a free action of the skin. This may be generally secured by small doses of antimonial wine with liquor ammoniæ acetatis, and the employment of the hot-air bath every night. It is well, too, to act on the bowels every second or third day by the use of compound jalap powder. Diluents, such as barley-water, toast-and-water, should be freely given. If there be much fever, and a tolerably firm pulse at the onset of the renal symptoms, cupping to the loins will probably be of service, and in any case dry cupping will do good. As the fever subsides, if the urine be tolerably abundant and of good specific gravity, but still contain albumen, the use of benzoate of ammonia, in five grain doses, seems to be of use; or if this fail, the tincture of the sesquichloride of iron will generally be of use. Sometimes it will be necessary to suspend these remedies, owing to an increase in the albumen and a smoky color of the water with a scantiness in amount, and to return to the hot-air baths and diaphoretics. The only diuretics besides benzoic acid that are allowable in this affection are, I believe, diluent drinks and digitalis, which appear to be of service when the pulse is quick and heat of skin considerable. Never rest satisfied or think your patient out of danger so long as albumen or still more blood remains in the urine; at any time while this lasts, one of the secondary inflammations or convulsions may set in, and rapidly carry him off.

If convulsions occur, and the patient be not previously very anæmic, bloodletting must be resorted to once and to a good quantity. This may be followed up by dry-cupping to the loins, hot-air baths, and compound jalap powder or elaterium. Trousseau recommends large blisters to the legs; most English authors forbid blistering in any shape, from the fear that cantharides will act injuriously on the already impaired kidneys. They have been so frequently used beneficially, that this theoretical objection to their employment need not, I think, have much weight. I am not aware that their use has ever been followed by increase of the renal symptoms.

If pleurisy set in, or pneumonia, I advise cupping, with or without the knives, according to the force of the pulse; and the subsequent cautious employment of calomel in small frequently-repeated doses. Here, again, I run counter to a commonly-received doctrine that calomel should not be given in albuminuria; my experience, however, inclines me to believe that in the early periods of nephritis, before renal degeneration has much advanced, there is no risk, but positive benefit from its use. If there be much restlessness or distress, the calomel should be combined with Dover's powder. At the same time liq. ammoniæ acetatis and, if the pulse be firm, small doses of antimonial wine may be given. In peritonitis, I believe that opium, with very small doses of mercury, is the sheet anchor. In one very severe case, in which a lady had scarlatina immediately after parturition, followed by peritonitis, the recovery appeared to be entirely due to the use of opium in full doses, combined with small doses of gray powder, until the gums were slightly affected.—*Medical Times and Gazette.*

